# Proteomics-Based Investigation of Sexual Dimorphism in Swim Bladder Texture of Chu’s Croaker (*Nibea coibor*)

**DOI:** 10.3390/foods14091586

**Published:** 2025-04-30

**Authors:** Haoran Zhang, Jiali Lin, Haoji Guo, Xianda He, Wanying Jiang, Lin Yan, Kuoqiu Yan, Xiaobo Wen, Fan Lin

**Affiliations:** 1 College of Marine Sciences, South China Agricultural University, Guangzhou 510642, China; 2 Agro-Tech Extension Center of Guangdong Province, Guangzhou 510520, China; 3 Guangdong Beluga Whale Marine Biotechnology Co., Ltd., Huizhou 516300, China; 4 Guangdong Provincial Key Laboratory of Marine Biotechnology, Institute of Marine Sciences, Shantou University, Shantou 515063, China

**Keywords:** swim bladder, texture, collagen fiber, proteomic, collagen XII

## Abstract

The swim bladder of Chu’s croaker (*Nibea coibor*) is an expensive food with high texture requirements. In this study, we found that male swim bladder had better toughness than female. To explore the main determining factor affecting texture properties of swim bladder, a comparison of proximate composition, collagen microstructure, and proteomics was carried out between male and female swim bladders. Results indicated that it should be collagen microstructure mainly affecting the texture characteristics of swim bladder, rather than the composition. The collagen fibers in male swim bladder were significantly more uniform and regular than female. By proteomics analysis, it was further discovered that collagen XII was the most significantly up-regulated protein in the male swim bladder and may be crucial in morphology of collagen fibers. In summary, collagen XII was identified to be a potential key molecule affecting the texture of the swim bladder, mainly through regulating properties of collagen fibers. This study contributes to a deeper understanding of the structural characteristics of swim bladder collagen and provides critical molecular targets for the regulation of texture in swim bladder.

## 1. Introduction

Food palatability is determined by human perception such as texture, flavor, appearance, sound, and temperature [1]. Among those factors, texture play a pivotal role, especially for aquatic products. Texture refers to a person’s sensory perception of a product, including their stress response and tactile characteristics [2]. It is usually measured or presented in the form of mechanical properties such as hardness, gumminess, resilience, cohesiveness, springiness, adhesiveness, and viscosity [3]. The factors influencing the texture characteristics of aquatic products are multifaceted, encompassing physical attributes (such as species, age, size, and microstructure), chemical properties (such as moisture content and distribution, fat content and distribution, and collagen content), as well as processing methods (such as storage conditions, freezing, refrigeration, high-pressure treatment, salting, and smoking), among other dimensions [4]. To date, the major determining factor for texture trait may vary among tissues, depending on the specific physiological structure and biochemical components.

Collagen is the most abundant protein in the fish swim bladder, constituting over 70% of its dry weight. Collagen fibers composed of type I collagen serve as a crucial structural framework that maintains the mechanical support of the swim bladder [5]. Collagen fibers, formed through tight alignment and aggregation of collagen molecules, are widely distributed in the connective tissues of animals. Each collagen molecule consists of three polypeptide chains characterized by a glycine residue at every third amino acid position, typically following a Gly-X-Y repeating pattern, where X and Y can represent any amino acids but are predominantly proline and hydroxyproline [6]. This distinctive repeating sequence facilitates the formation of a rigid right-handed triple helix structure through tight coiling of the three polypeptide chains, endowing collagen with exceptional mechanical strength, chemical stability, and exceptionally high tensile resistance in fibrillar assemblies [7,8]. The tissue-specific variations in collagen fibers demonstrate their adaptability to distinct biomechanical environments. For instance, in tendons, which are subjected to high tensile forces, collagen fibers exhibit larger diameters and a tightly packed arrangement, whereas those in more elastic tissues like skin adopt flexible and finer configurations. Such structural diversity and adaptability underscore the indispensable role of collagen fibers in preserving organismal structural integrity and facilitating specialized physiological functions [9,10].

*N. coibor* is a marine economic Sciaenoid fish that is highly sought after by consumers, attributed to its exceptional nutritional profile, delicate flavor, and large swim bladder rich in collagen [11]. The swim bladder, known as fish maw, has significant economic value, exceeding 12,000 RMB/kg in coastal regions of China [12]. Interestingly, sexual dimorphism in the textural quality of swim bladder from Sciaenidae fish has been generally recognized by consumers, with better quality of swim bladder from male fish [13]. In some Sciaenid species, the market price of male swim bladders is more than twice that of females [14]. Therefore, investigating the sexual dimorphism in the texture of Sciaenid swim bladders can provide a scientific basis for the development of high-quality swim bladder products, which is of significant importance for the fish maw industry. So far, however, little has been known concerning the major determining factor underlying the different texture quality of swim bladder. In this study, we investigated the sexual dimorphism in the texture of the *N. coibor* swim bladder and the underlying causes of its formation. Furthermore, through comparative proteomic analysis, we preliminarily explored the molecular mechanisms responsible for the differences in swim bladder texture between males and females.

## 2. Materials and Methods

### 2.1. Experimental Fish and Tissue Collection

Twenty adult *N. coibor* (1956.30 ± 157.00 g) of the same fish age (same hatching batch) were obtained from a local commercial farm in Rao Ping, Guangdong, China. The fishes were humanely euthanized in pH-buffered tricaine methanesulfonate (250 mg/L) (Augsburg, Germany), and their sex was confirmed by the examination of gonadal morphology. The swim bladder were harvested from five male (MSB) and five female (FSB) individuals for further analysis. For texture analysis, two pieces of swim bladder sample (middle dorsal part, 2 cm × 2 cm) were taken from each fish, stored on ice, and immediately subjected to evaluation. Additionally, swim bladder tissues were preserved at −80 °C for proximate composition and collagen content analysis. For histological examination, swim bladder samples were fixed in 4% paraformaldehyde in phosphate-buffered saline (PBS). For transmission electron microscopy analysis, samples were fixed in 2.5% glutaraldehyde in 0.1 M phosphate buffer (PH 7.0–7.5). Finally, proteomic profiling was performed on three adult male and three adult female swim bladder samples.

### 2.2. Textural Properties Analyses of Swim Bladder

Swim bladder samples (2 cm × 2 cm), stored at 4 °C and analyzed within 24 h of collection, were subjected to a texture analysis using a Texture Analyzer (Universal TA, Shanghai Teng Ba Instrument Technology Co., Ltd., Shanghai, China). The TPA (Texture Profile Analysis) model was employed with a cylindrical probe to determine the textural properties as described previously [13], including springiness, shear force, gumminess, chewiness, and hardness. The type of measurement was pushed down, and the deformation percentage was set at 20%. Each sample piece underwent five probing cycles with an interval of 30 s between each cycle.

### 2.3. Section and Masson Staining

The swim bladder samples, which were preserved in 4% paraformaldehyde in PBS, underwent routine processing for paraffin embedding and sectioning. The resulting paraffin sections, with a thickness of 4 μm, were subjected to dewaxing and rehydration procedures before being stained using the Masson kit (Servicebio, Wuhan, China). Subsequently, all sections were examined using an upright optical microscope (Nikon, Tokyo, Japan).

### 2.4. Transmission Electron Microscopy (TEM)

The microstructure of the swim bladder was analyzed using a transmission electron microscope (JEOL JEM 2010, Tokyo, Japan) at 160 kV. The swim bladder samples underwent rinsing with 0.1 M sodium parahydroxybenzoate buffer (pH 7.4) for 1 h, followed by fixation in 1% osmium solution and 2% uranyl acetate for 1 h. Subsequently, the samples were dehydrated in a series of ethanol solutions (70%, 80%, 90%, and 100%) and embedded in Epone resin. Thin sections of 80 nm thickness were then cut using an LKB ultramicrotome, deposited on copper grids, stained with 1% uranyl acetate, and photographed. TEM images of swim bladders were randomly selected and the roundness and number of collagen fibers within a 1 μm^2^ area in the up, down, left, and right directions of the image were counted. The roundness of the collagen fibrils was calculated with the following criterion: a value of 1 denoted a perfectly rounded contour and values <1 denoted a progressive departure from the circular contour. During collagen fiber density counting, fibers partially within the 1 μm^2^ area are still counted as one.

### 2.5. Proximate Composition and Collagen Content Analysis

Crude protein, moisture content, and crude lipid content of the swim bladder were analyzed using standard methods [15]. Crude protein content was determined by the Kjeldahl method using a Kjeldahl Auto Sampler System 1035 Analyzer (Foss, Hoganas, Sweden), with nitrogen conversion factor (6.25) applied. Crude lipid content was evaluated by ether extraction using a Soxtec TM 8000 extraction system (Foss, Hoganas, Sweden). Moisture content was determined by oven drying at 105 °C for 6 h using an FUMA DGX-8053B drying oven (Shanghai, China).

### 2.6. Collagen Content Analysis

Collagen content was determined using Hydroxyproline Assay Kit (No. A030-2-1, Nanjing Jiancheng Bioengineering Institute, Nanjing, China). Following the manufacturer’s instructions: swim bladder samples were hydrolyzed with the NaOH-containing hydrolyzation buffer at 95 °C for 20 min. Then, the pH was adjusted to 6.0–6.8 with reagents (pH adjusting liquid A and B) provided in the kit. The mixture was then diluted by double-distilled water and the supernatant was collected after carbon adsorption and centrifugation. The diluted samples, ddH_2_O (negative control) and standard Hyp (5 μg/mL, positive control) were mixed with reagent I (2:1) and incubated for 10 min or at room temperature. Then, reagent II (equivalent in re-agent I) was added and left to stand for 5 min at room temperature. Finally, the above mixture was mixed with reagent III and incubated in 60 °C water for 15 min. The Hyp concentration was determined by colorimetry at OD 560 nm after centrifugation.

### 2.7. Proteomics Analysis

This study used iTRAQ labeling and a high-resolution mass spectrometry system to ensure the reproducibility and reliability of proteomics. Proteomic profiling procedures encompassed three key stages as detailed below [16]: (1) Sample preparation: put the swim bladder samples into a centrifuge tube containing protein lysate (6 M CH_6_ClN_3_, 500 mM C_7_H_17_NO_3_ and 0.1% Triton X-100), and use a homogenizer to make a homogeneous protein samples; (2) Protein labeling: after detecting the protein concentration, 100 μg protein sample was labeled by iTRAQ assay (Thermo Fisher Scientific, Waltham, MA, USA); (3) Protein separation and identification: the labeled protein samples were separated using a nano-high-performance liquid chromatography system (Dionex, Sunnyvale, CA, USA) (Protein separation is carried out through SCX chromatography column, and then protein identification is carried out through Pico-Frit chromatography column and nano-ESI-QqTOF system in series), analyzed using Analyst QS version 1.1 software, and compared to the UniProtKB (http://www.uniprot.org, accessed on 17 August 2022) database to annotate the identified proteins. Comparing the swim bladder of male and female *N. coibor*, proteins showing at least twofold differential expression (ratio < 0.5 or >2) with *p* < 0.05 were selected and further analyzed.

### 2.8. Statistics

The data were presented as mean ± SD, number of replicates *n* (biological replicates) is indicated in the figure legends. Normal distribution was confirmed using the Shapiro–Wilk test for all variables. Levene’s test was used to assess homogeneity of variance. Statistical analysis was conducted using SPSS 20.0, and Student’s *t*-test was applied. A significance level of *p* < 0.05 was utilized.

## 3. Results and Discussion

### 3.1. Texture Quality Between MSB and FSB

**Figure 1:**
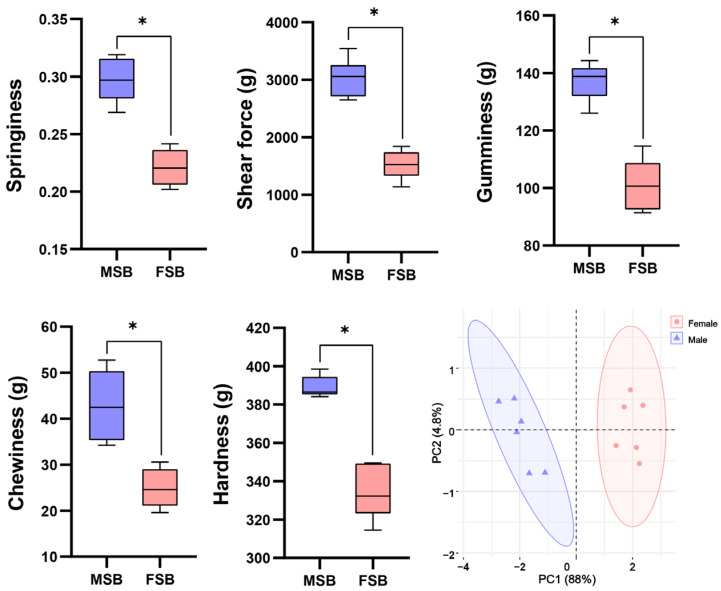
Sexual differences in textural quality of swim bladder. Data are shown as mean ± S.D (*n* = 5). Statistical analyses were performed using Student’s *t*-test, * *p* < 0.05.

The texture difference between MSB and FSB was shown in Figure 1. Compared with FSB, all the tested textural parameters (hardness, springiness, gumminess, chewiness, and shear force) were higher in MSB (*p* < 0.05). PCA analysis indicated distinct separation of the textures of MSB and FSB, which represent the differences in texture properties of MSB and FSB. Obviously, the MSB has better toughness. Sexual dimorphism in fish is widely observed in nature, with most studies focusing on external morphology and growth rates. For instance, male tilapia exhibit significantly faster growth rates compared to females [17], whereas in flatfish, the opposite pattern is observed [18]. Sexual dimorphism primarily involves two key mechanisms: first, natural selection may preserve a significant advantage in specific traits of one sex, resulting in the expression of sexual dimorphism even during early life stages; second, sex-related physiological mechanisms can indirectly influence these traits, thereby inducing sexual dimorphism during the process of sexual maturation [19]. Sciaenidae species can make sounds through the vibration resonance of their swim bladder. Due to gender-related behaviors such as courtship, the frequency and intensity of vocalizations of male Sciaenidae are significantly stronger than the females, indicating that male swim bladder may be subject more substantial mechanical stimulation [20]. We speculate that this may be related to the sexual dimorphism of the swim bladder texture, which warrants further investigation.

### 3.2. Proximate Composition and Collagen Content Between MSB and FSB

**Figure 2:**
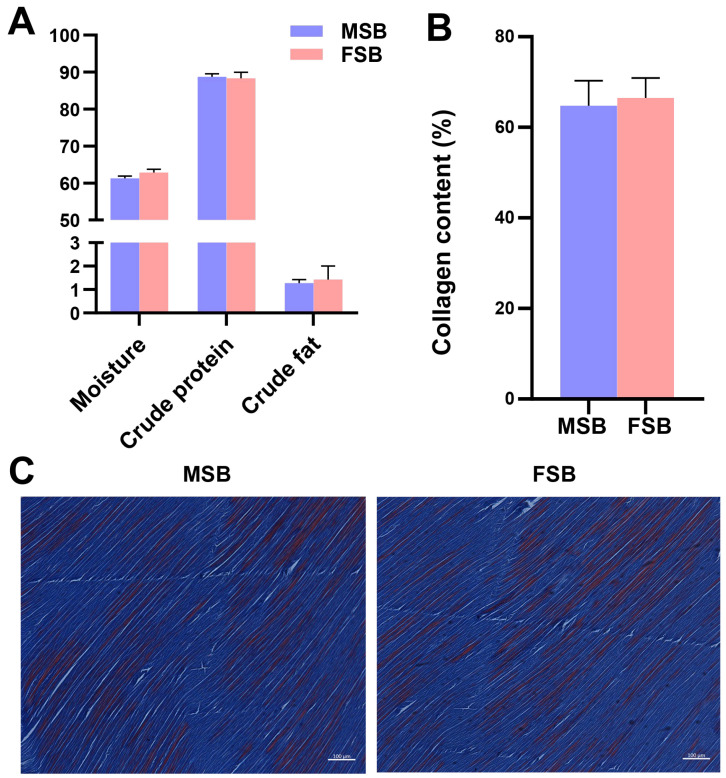
Sexual differences in composition of swim bladder. (**A**) Proximate composition of MSB and FSB. (**B**) Collagen content of MSB and FSB. (**C**) Masson staining of MSB and FSB. Data were shown as mean ± S.D (*n* = 5). Statistical analyses were performed using Student’s *t*-test.

Under the same external conditions, the content and distribution of the major composition (fat, carbohydrate, protein) are one of the important factors affecting tissue texture [21]. However, despite significant differences in the texture of MSB and FSB, no significant differences in the crude composition were noticed (Figure 2A). Notably, the swim bladder of male fish exhibits a slightly lower moisture content and a slightly higher crude protein content compared to that of females, suggesting that male swim bladder may possess a higher proportion of dry matter. The amino acid composition of MSB and FSB is shown in Table 1. Gly is the amino acid residue with the highest content, 15.93% and 15.64%, respectively, showing typical collagen characteristics. Gly represents the most abundant amino acid in collagen molecules, and its repetitive Gly-X-Y constitutes a critical molecular structural characteristic of collagen [6]. But the individual amino acid residues of MSB and FSB also showed insignificant differences. Collagen is the most important structural protein in the extracellular matrix. Many studies have revealed that collagen is essential for maintaining the tensile strength and structural integrity of tissues [22]. Most studies show a positive relationship between collagen content and tissue toughness [23]. But similarly, we found no significant differences in the collagen content of swim bladders between sexes (Figure 2B). This finding was further supported by Masson’s staining of the swim bladder sections (Figure 2C). Through Masson’s staining, the swim bladders of different gender were very similar. Based on the aforementioned findings, although the swim bladder may contain other undetected trace components, our results suggest that the compositional differences are unlikely to be the primary factor contributing to the textural variation between female and male swim bladders.

### 3.3. Microstructures Between MSB and FSB

**Figure 3:**
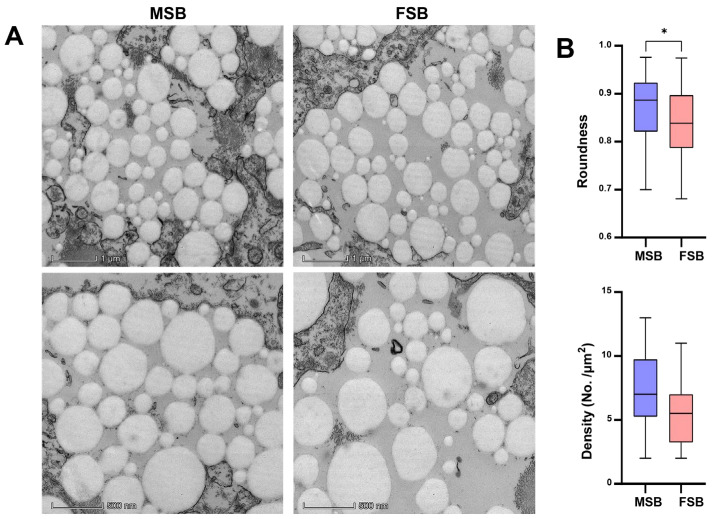
Sexual differences in microstructure of swim bladder. (**A**) TEM observation of MSB and FSB. (**B**) Collagen fiber roundness and density of MSB and FSB. Data were shown as mean ± S.D (*n* (roundness) = 100; *n* (density) = 20). Statistical analyses were performed using Student’s *t*-test, * *p* < 0.05.

**Figure 4:**
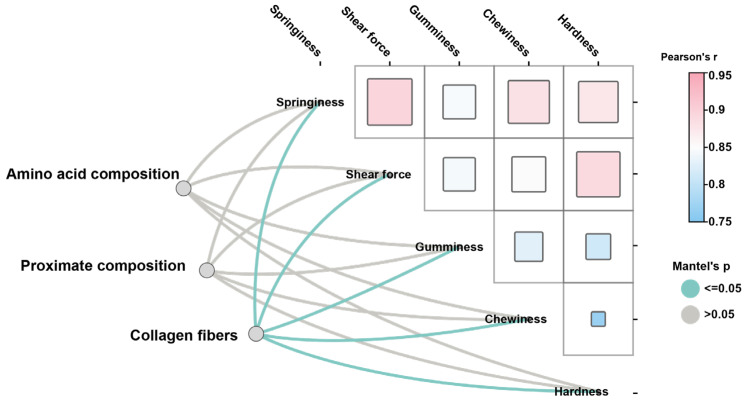
Mantel test analysis of amino acid composition, proximate components, and collagen fiber morphology on swim bladder texture. Mantel test *p* ≤ 0.05 indicates significant correlation.

Interestingly, TEM analysis of the swim bladder showed obvious differences in the morphology of collagen fibers (Figure 3A). The collagen fibers in MSB were significantly more regular than in FSB (*p* < 0.05). In addition, the density of collagen fibers in MSB was also higher than that in FSB (Figure 3B). Therefore, although collagen content was similar, a discernible difference in the microstructure of collagen fibers was demonstrated between MSB and FSB. Collagen fibers constitute the fundamental building blocks of skin, bone, tendons, ligaments, and a variety of other tissues and organs, serving as one of the critical components in maintaining the structural integrity and functionality of the organism, and its primary role is to confer mechanical strength and support to tissues [24]. The alterations in collagen fiber properties, such as fibril size, arrangement, and cross-links, can also significantly affect tissue textures. For instance, during bovine semitendinosus development, the increasing consistency in collagen fiber arrangement is directly correlated with rising skeletal muscle toughness [25]. Similarly, researchers found that collagen cross-linking and fiber stability, rather than collagen content, have a more significant effect on bovine muscle texture [26]. Octopus has a higher muscle texture than guitarfish and cazon also due to a higher degree of cross-linking of collagen fibers [27]. Fortunately, as demonstrated above, we found that the microstructure of collagen fibers in the *N. coibor* swim bladder showed significant gender differences. The Mantel test is a statistical method used to assess the correlation between two distance (or similarity) matrices. Although the accuracy of the Mantel test analysis may be unstable with small sample sizes, we still performed correlation analyses using the Mantel test by treating swim bladder texture and their potential influencing factors (proximate components, amino acid composition, and collagen fiber morphology) as separate distance matrices. The results show that the shape and density of collagen fibers were significantly correlated with the texture of the swim bladder, rather than the crude or amino acid composition of swim bladder (Figure 4). Therefore, the difference in physical characteristics of collagen fibers may affect the texture quality of swim bladder.

### 3.4. Proteomic Analysis of MSB and FSB

**Figure 5:**
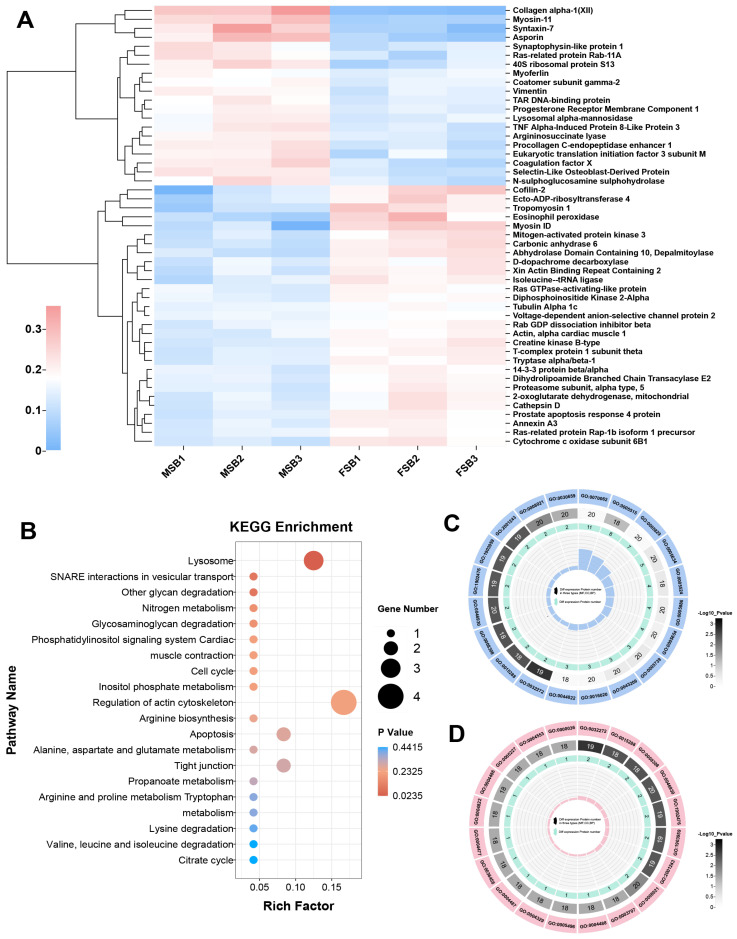
Transcriptome analysis of swim bladders of different sexes. (**A**) Heat map of DEPs. Red for up-regulated and blue for down-regulated. (**B**) Statistics of KEGG pathway enrichment of DEPs. (**C**) Statistics of GO pathway enrichment of DEPs (number of DEPs). (**D**) Statistics of GO pathway enrichment of DEPs (significance of the GO pathway).

Our results demonstrated that the major sexual difference in swim bladder collagen is related to the structure of collagen fiber. To investigate the molecular mechanism underlying this difference, comparative proteomic analysis of MSB and FSB was conducted. In the proteomic analysis, a total of 952 proteins were annotated, including 471 in the clusters of orthologous groups of proteins (COG) database (Figure S1), 868 in the gene ontology (GO) database (Figure S2), and 512 in the kyoto encyclopedia of genes and genomes (KEGG) database (Figure S3). Most of these proteins are related to protein folding, modification, and transport. Through further comparison of the proteomics, we screened out 49 differentially expressed proteins (DEPs) in female and male swim bladders (Figure 5A). These DEPs were subjected to enrichment analysis of the KEGG pathway database and the GO pathway database, respectively. The KEGG pathway enrichment analysis is a bioinformatics-based methodology designed to identify significantly enriched biological pathways from high-throughput omics data (e.g., transcriptomic, proteomic, or metabolomic datasets), thereby elucidating potential functional mechanisms involving differentially expressed genes, proteins, or metabolites [28]. In the KEGG enrichment pathway analysis, the pathway with the highest significance is “Lysosome”, and the pathway with the highest rich factor is “Regulation of actin cytoskeleton” (Figure 5B). Both pathways are involved in the regulation of the extracellular matrix. Studies have shown that lysosomes regulate collagen protein structure through enzymatic degradation (e.g., cathepsins) and autophagy-mediated extracellular matrix remodeling, with dysfunctions in lysosomal activity linked to collagen accumulation in fibrotic diseases [29]. The regulation of the actin cytoskeleton modulates collagen protein synthesis, alignment, and fibril stability through the regulation of cellular mechanical tension, thereby orchestrating the structural remodeling of the extracellular matrix [30]. “Lysosomes” and “regulation of the actin cytoskeleton” may be potential pathways for the swim bladder to regulate tissue mechanical properties. Gene ontology (GO) pathway enrichment analysis serves as a methodological approach to identify significantly overrepresented biological processes, molecular functions, and cellular components within gene expression datasets, thereby facilitating the interpretation of the biological significance underlying gene expression patterns under specific experimental conditions [31]. Using the number of DEPs in the GO pathway as the screening criterion (Figure 5C), the GO with the most enriched DEPs is GO:0070062 (extracellular exosome). And using the significance of the GO pathway as the screening criterion (Figure 5D), the most significant GO is GO:0032272 (negative regulation of protein polymerization).

**Figure 6:**
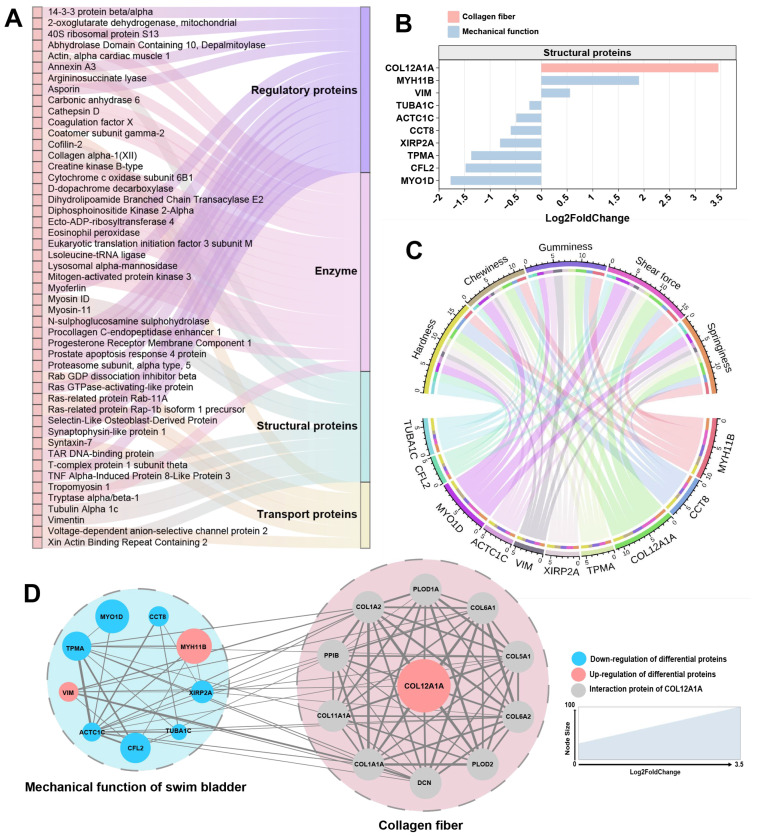
Analysis of DEPs associated with differences in swim bladder collagen. (**A**) Classified DEPs according to enzymes, structural proteins, transport proteins, and regulatory proteins. (**B**) Log2FoldChange and functions of structural proteins in DEPs. (**C**) Pearson correlation analysis of structural proteins in DEPs and swim bladder texture. The larger the scale of DEPs, the higher correlation with texture. (**D**) Protein interaction network of structural proteins in DEPs. The larger the size of the node shape, the higher the Log2FoldChange of protein. Red for up-regulated and blue for down-regulated.

According to the function of the protein, these DEPs are divided into four categories: enzymes, structural proteins, regulatory proteins, and transport proteins (Figure 6A). Structural proteins are a category of proteins that play crucial roles in providing support, shape, and strength to cells and tissues in living organisms. Therefore, changes in the properties of collagen fibers in fish bladder species may be related to changes in certain structural proteins [32]. In the structural proteins of DEPs, we identified two types: collagen XII (a fibril-associated collagen with interrupted triple helices (FACIT)) and actin/myosin (along with related regulatory proteins), linked to mechanical properties. Among these structural proteins, collagen XII exhibited the most significant differences (Figure 6B), showing a marked upregulation in MSB compared to FSB. In the correlation analysis between structural proteins and swim bladder texture, collagen XII also displayed the strongest correlation (Figure 6C). Collagen XII is a fibril-associated collagen, widely expressed in bone, ligaments, tendons, fibrocartilage, smooth muscle, skin, and articular cartilage [33]. It is a homotrimer with two collagenous domains (COLI and COLII) and three noncollagenous regions (NC1, NC2, and NC3) [34]. Collagen XII is codistributed with collagen I and collagen II, and it changes the properties of collagen fibers by binding to collagen fibrils via its collagenous domain [35]. Despite its inability to independently establish collagen fibers, collagen XII plays a pivotal role in regulating and packaging collagen fibers [36]. Our study revealed significant differences in collagen fiber morphology between MSB and FSB, potentially attributable to the differential expression of collagen XII. This finding is supported by similar results observed in collagen XII knockout mouse skin, where collagen fibers were thicker and more irregular, in contrast to thinner and more regular fibers in collagen XII-overexpressing mouse skin [37]. The same results were observed in the skin of EDS patients with decreased collagen XII levels [38]. It is worth noting that collagen XII is typically expressed at higher levels in tissues with mechanical functions, showing a significant positive correlation between its expression and mechanical stress within the tissue [39,40,41,42]. In the cornea, a lack of collagen XII make the tissue stiffer and less elastic [43]. Excess collagen XII expression is also associated with hardened skin in patients with scleroderma [44]. The swim bladder is a tissue that frequently undergoes mechanical activity (contract and relax frequently to regulate buoyancy). It is interesting to note that the collagen fibers are more regular, uniform, and neatly arranged in male swim bladders with higher expression level of collagen XII. At the same time, increased texture properties (hardness and toughness) were also observed in the male swim bladder. This seems to be consistent with most known functions of collagen XII. Through protein–protein interaction network analysis using the STRING database (https://cn.string-db.org, accessed on 9 May 2023), a strong connection was identified between mechanically functionally relevant DEPs and collagen XII (and its interacting proteins) (Figure 6D). This finding suggests a potential regulatory mechanism between collagen XII and the mechanical properties of swim bladder, indicating that collagen XII may serve as a key regulatory target for modulating swim bladder texture. Overall, the morphological alterations in collagen fibers mediated by collagen XII may be the primary factor contributing to the sexual dimorphism in the texture of the *N. coibor* swim bladder. Our research provides an important theoretical foundation for subsequent production of a swim bladder with superior textural characteristics.

## 4. Conclusions

The texture of male and female swim bladders exhibits pronounced sexual dimorphism. Compared with the female swim bladder, the males have greater toughness, which may be mainly caused by the more uniform shape and higher density of collagen fibers. Proteomic analysis further revealed that the highly expressed collagen XII in the male swim bladder is likely the primary factor contributing to sexual dimorphism in swim bladder texture. Collagen XII can bind to collagen fiber via its collagenous domains, thereby affecting the morphology of swim bladder collagen fiber. This study provides novel insights into the sexual dimorphism of swim bladder texture in Sciaenidae fish, offering a potential regulatory target for texture of the swim bladder, which holds scientific significance for the fish maw industry and Sciaenidae aquaculture.

## Figures and Tables

**Table 1:** Amino acid composition of MSB and FSB.

Amino Acid (%)	MSB	FSB
Arg	6.12 ± 0.62	6.76 ± 0.78
His	0.54 ± 0.04	0.59 ± 0.06
Lys	1.52 ± 0.10	1.68 ± 0.19
Gly	15.93 ± 0.86	15.64 ± 0.65
Ser	1.72 ± 0.04	1.80 ± 0.05
Cys-Cys	0.03 ± 0.00	0.03 ± 0.01
Asp	4.02 ± 0.10	3.87 ± 0.53
Glu	8.09 ± 0.37	8.24 ± 0.41
Ala	7.75 ± 0.25	7.63 ± 0.15
Thr	2.02 ± 0.12	2.09 ± 0.10
Pro	7.94 ± 0.36	7.89 ± 0.18
Met	1.63 ± 0.17	1.27 ± 0.80
Val	2.26 ± 0.16	1.88 ± 0.79
Tyr	0.73 ± 0.05	0.65 ± 0.13
Ile	0.82 ± 0.06	0.82 ± 0.15
Leu	1.85 ± 0.09	1.77 ± 0.47
Phe	1.63 ± 0.14	1.67 ± 0.22
Hyp	4.09 ± 0.09	4.52 ± 0.18
Imino acid	12.02 ± 0.27	12.42 ± 0.21

Note: Data are shown as mean ± S.D (*n* = 5). Statistical analyses were performed using Student’s *t*-test.

## Data Availability

The original contributions presented in this study are included in the article/Supplementary Materials. Further inquiries can be directed to the corresponding authors.

## Supplementary Materials

The following supporting information can be downloaded at: https://www.mdpi.com/article/10.3390/foods14091586/s1, Figure S1: COG function classification; Figure S2: GO function classification; Figure S3: KEGG function classification.
