# Supplementary material for: Proteomics-Based Investigation of Sexual Dimorphism in Swim Bladder Texture of Chu’s Croaker (Nibea coibor)
Source: Foods. 2025 Apr 30;14(9):1586. doi: 10.3390/foods14091586 (PMC12071268; doi:10.3390/foods14091586)
Supplement: Supplementary file 1 [file foods-14-01586-s001.zip › Figure S2.pdf]

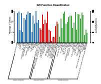
GO Function Classification
