# Supplementary figures and images for: Proteomics-Based Investigation of Sexual Dimorphism in Swim Bladder Texture of Chu’s Croaker (Nibea coibor)
Source: Foods. 2025 Apr 30;14(9):1586. doi: 10.3390/foods14091586 (PMC12071268; doi:10.3390/foods14091586)

Figure 1: Histogram of the number of occurrences of each word in the corpus.

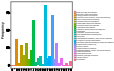

Supplement: Supplementary file 1 [file foods-14-01586-s001.zip › Figure S1.pdf]

Weekly Revenue Breakdown

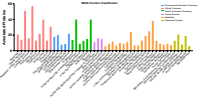

Supplement: Supplementary file 1 [file foods-14-01586-s001.zip › Figure S3.pdf]
